# Microarray analysis identifies IL-1 receptor type 2 as a novel candidate biomarker in patients with acute respiratory distress syndrome

**DOI:** 10.1186/s12931-015-0190-x

**Published:** 2015-02-21

**Authors:** Melissa A Kovach, Kathleen A Stringer, Rachel Bunting, Xiaoying Wu, Lani San Mateo, Michael W Newstead, Robert Paine, Theodore J Standiford

**Affiliations:** Department of Internal Medicine, Division of Pulmonary and Critical Care Medicine, University of Michigan Medical Center, 109 Zina Pitcher Place, 4062 BSRB, Ann Arbor, MI 48109-2200 USA; Department of Clinical, Social and Administrative Sciences, University of Michigan College of Pharmacy, University of Michigan, Ann Arbor, MI USA; Center for Computational Medicine and Bioinformatics, University of Michigan Medical School, Ann Arbor, MI USA; Department of Immunobiology, Centocor Research & Development Inc., Radnor, PA USA; Department of Internal Medicine, Division of Pulmonary and Critical Care Medicine, University of Utah Medical Center, Salt Lake City, Utah USA

**Keywords:** Acute respiratory distress syndrome, Alveolar macrophage, Innate immunity, Microarray

## Abstract

**Background:**

Acute respiratory distress syndrome (ARDS) is a disease associated with a high mortality rate. The initial phase is characterized by induction of inflammatory cytokines and chemokines and influx of circulating inflammatory cells, including macrophages which play a pivotal role in the innate and adaptive immune responses to injury. Growing evidence points to phenotypic heterogeneity and plasticity between various macrophage activation states.

**Methods:**

In this study, gene expression in alveolar macrophages and circulating leukocytes from healthy control subjects and patients with ARDS was assessed by mRNA microarray analysis.

**Results:**

Both alveolar macrophages and circulating leukocytes demonstrated up-regulation of genes encoding chemotactic factors, antimicrobial peptides, chemokine receptors, and matrix metalloproteinases. Two genes, the pro-inflammatory S100A12 and the anti-inflammatory IL-1 decoy receptor IL-1R2 were significantly induced in both cell populations in ARDS patients, which was confirmed by protein quantification. Although S100A12 levels did not correlate with disease severity, there was a significant association between early plasma levels of IL-1R2 and APACHE III scores at presentation. Moreover, higher levels of IL-1R2 in plasma were observed in non-survivors as compared to survivors at later stages of ARDS.

**Conclusions:**

These results suggest a hybrid state of alveolar macrophage activation in ARDS, with features of both alternative activation and immune tolerance/deactivation.. Furthermore, we have identified a novel plasma biomarker candidate in ARDS that correlates with the severity of systemic illness and mortality.

**Electronic supplementary material:**

The online version of this article (doi:10.1186/s12931-015-0190-x) contains supplementary material, which is available to authorized users.

## Introduction

Acute respiratory distress syndrome (ARDS) is a deadly disease, with an incidence of roughly 200,000 cases per year in the United States and a mortality rate of approximately 40% [1]. ARDS is characterized by marked hypoxemia (PaO_2_/FiO_2_ < 300), diffuse bilateral infiltrates, and decreased lung compliance either due to direct injury (e.g., pneumonia, aspiration) or indirect injury (e.g., sepsis, pancreatitis, transfusion-related acute lung injury) [2-4]. Following the initial injury, a stereotyped process of tissue injury, inflammation, and alveolar capillary damage evolves to a fibroproliferative phase [5]. During the inflammatory phase, resident immune cells in the lung express pro-inflammatory chemokines and cytokines, which in turn stimulates the influx of circulating inflammatory cells into the interstitium and alveolar spaces [4].

Macrophages play a pivotal role in the innate and adaptive immune responses during host defense and response to injury. Recent research has uncovered distinctly heterogeneous populations of macrophages, as well as plasticity between different macrophage phenotypes [6]. This phenotypic polarization of macrophages is believed to be a consequence of factors present within the cell’s microenvironment [7,8]. Several broad categories of distinct macrophage phenotypes have been described. Classically-activated macrophages (M1) are generally associated with an inflammatory environment and mediate microbial phagocytosis, pro-inflammatory cytokine expression, and cellular immune responses [6,9,10]. Alternatively-activated macrophages (M2) are anti-inflammatory in nature, and are associated with tissue repair and humoral immune responses [9-11]. A third macrophage phenotype, referred to deactivation or immunoparalysis, can be induced by repeated exposure to various pathogen-associated molecular patterns (PAMPs) in-vitro (referred to as immune tolerance) or during the systemic inflammatory response in-vivo [12-14]. Macrophage deactivation is characterized by suppression of pro-inflammatory cytokines (e.g., TNF-α, IL-6, nitric oxide synthase-2) and enhanced expression of anti-inflammatory molecules (IL-10), and this phenotype has been described in circulating blood monocytes isolated from critically ill patients, including patients with sepsis [12,15-19]. Recent evidence also points to the presence of phenotypic heterogeneity, with a spectrum between classically activated, alternatively activated, and deactivated macrophages, particularly in the setting of chronic inflammatory states [10,20-22].

The identification of biomarkers correlating to disease activity in ARDS is an important goal, potentially allowing for earlier diagnosis and treatment, as well as providing prognostic information [23]. Nevertheless, successful association of biomarkers to disease severity remains elusive. S100 calcium binding protein A12 (S100A12) is a known chemoattractant, and is a pro-inflammatory ligand for the RAGE receptor [24]. Previous studies have found that S100A12 protein is upregulated in both the bronchoalveolar lavage (BAL) fluid and plasma of patients with ARDS [25-27]. However, these studies did not assess whether S100A12 levels correlated with indices of ARDS severity. Conversely, the anti-inflammatory IL-1 decoy receptor, IL-1R2, has recently been shown to be elevated in plasma during sepsis [28], as well as a mouse model of chemically-induced lung injury [29]. However, there have been no studies examining the association of IL-1R2 and human lung disease, specifically ARDS.

In this pilot study, we analyzed gene expression profiles in isolated alveolar macrophages (AM) obtained from BAL samples and buffy coat leukocytes isolated from patients with ARDS. As compared to cells from healthy subjects, there was considerable over-representation of immune activation genes, including genes involved in leukocyte trafficking, phagocytosis, microbial killing, apoptosis and lymphocyte activation. We also found a gene expression profile in AM with features of both alternative macrophage activation and immune tolerance/deactivation, suggesting the presence of unique macrophage phenotypes present early in the course of ARDS that may have important functional significance in the pathogenesis and course of disease in this patient population. Furthermore, we assessed plasma and BAL levels of two of the most highly induced genes identified in our microarray analysis, S100A12 and IL-1R2. While both analytes were significantly up-regulated in both the plasma and BAL fluid of ARDS patients as compared with healthy control subjects, only plasma levels of IL-1R2 correlated significantly with APACHE III scores and mortality. We have therefore identified a novel plasma biomarker candidate for ARDS, which may aid in early diagnosis and which carries prognostic implications for disease severity in ARDS.

## Materials and methods

### Patient selection

Patients with ARDS that were enrolled in the Acute Lung Injury Specialized Center of Clinically Oriented Research randomized trial of granulocyte-macrophage colony stimulating factor conducted at the University of Michigan between July 2004 and October 2007 were studied [30]. Samples from eighteen patients were included in the microarray analysis, whereas samples from a larger group of patients were used for measurement of S100A12 and IL-1R2 quantitation. Criteria for ARDS were as follows: Acute onset of illness with: 1) PaO_2_/FiO_2_ ≤ 300; 2) bilateral infiltrates consistent with pulmonary edema on frontal chest radiograph; 3) requirement for positive pressure ventilation via an endotracheal tube; 4) no clinical evidence of left atrial hypertension; 5) if measured, pulmonary arterial wedge pressure ≤ 18 mm Hg; and 6) the aforementioned criteria occurring together within a 24-hour interval. These subjects underwent serial bronchoscopy with BAL and peripheral blood collection at various time points post onset of ARDS. For the microarray analysis, a subset of 18 patients were studied. In this subset, sample collection was performed between days 0–4 from the time patients fulfilled the diagnostic criteria for ARDS [30] and only samples collected from patients randomized to the placebo control arm of the study or the observational study were utilized. Of the 18 patients enrolled in the microarray sub-study, 17 patients had blood samples drawn for buffy coat analysis, and 7 patients underwent bronchoscopy for the collection of BAL for AM analysis [30,31]. Serial blood and BAL samples were also collected from an additional 70 ARDS patients for a total of 88 samples analyzed for cytokine levels by ELISA. As with microarray samples, only patients from the observational or placebo arms were analyzed. A single blood and BAL sample were collected from 5 healthy subjects following informed s and under protocols approved by the University of Michigan Institutional Review Board, which served as controls for both the microarray and ELISA analyses. All control subjects were less than 55 years of age, on no medications, and were life-long non-smokers. Demographic and clinical data of the microarray study population are summarized in Table 1. Demographic and clinical data of the larger population are summarized in Table 2.

**Table 1:** **Demographic and clinical data of microarray study population**

	**Control**	**ARDS patients**
Age	45.8 ± 8.31	46.72 ± 15.62
Male:Female	3:2	12:6
APACHE Score		66.44 ± 13.12
P/f ratio		107.33 ± 50.78
Ventilator-Free Days		14.67 ± 8.0
Organ Failure-Free Days		18.16 ± 10.97
**Diagnosis**		
Pneumonia		6 (37.5%)
Aspiration		3 (18.75%)
Sepsis		7 (43.75%)
Pancreatitis		1 (6.25%)
Other		1 (6.25%)

**Table 2:** **Demographic and clinical data of study participants**

	**Control**	**ARDS patients**
Age	45.8 ± 8.31	47.2 ± 13.95
Male:Female	3:2	31:19
APACHE Score		58.12 ± 17.48
P/f ratio		128.4 ± 62.46
Ventilator-Free Days		12.26 ± 10.64
Organ Failure-Free Days		14.21 ± 12.52
**Diagnosis**		
Pneumonia		14 (29.2%)
Aspiration		7 (14.6%)
Sepsis		18 (37.5%)
Pancreatitis		4 (8.3%)
Other		5 (10.4%)

### Sample preparation

Peripheral whole blood samples were collected from ARDS patients and healthy controls into heparin-containing Vacutainer® tubes (Becton-Dickinson, Franklin, NJ, USA) and centrifuged (1500 rpm for 10 minutes at room temperature). The buffy coat cell layer was carefully aspirated and buffy coat cells were suspended in Trizol® reagent (Invitrogen; Grand Island, NY). Samples were stored at −20°C. The remaining plasma was stored at −80°C.

Bronchoalveolar lavage fluid was obtained from control subjects and ARDS patients by bronchoscopy using a standard technique as previously described [30,31]. The BAL was centrifuged (1500 rpm at 4°C for 10 minutes). Cell free supernantant was removed and stored (−70°C). Cells were resuspended and plated in plastic culture dishes containing media (RPMI, manufacturer, state, USA). After a one hour incubation (5% CO_2_, 37°C), non-adherent cells were removed by washing, and adherent macrophages were resuspended in Trizol® reagent and stored (−20°C). Cell differentials post adherence revealed >90% macrophages by morphology, and highly expressed both CD163 and CD14. The mRNA expression of CD163 and CD14 by AM isolated from ARDS patients was not different than that expressed by AM collected from control patients (data not shown).

### Microarray analysis

Frozen buffy coat and AM samples were shipped to Centocor (Radnor, PA).

The quantity and purity of RNA were measured using a Thermo Scientific NanoDrop 1000 Spectrophotometer (NanoDrop technologies, Berlin, Germany) while the quality of RNA was determined by running aliquots on the 2100 Bioanalyzer (Agilent Technologies, Waldbronn, Germany). Samples with a RNA integrity number <6.5 or a concentration <50 ng/μl were excluded from the study while samples with a RNA integrity number ≥6.5 and a concentration ≥50 ng/μl were labelled. TotalRNA was reverse transcribed and product cDNA amplified, fragmented, and labelled using the Ovation RNA Amplification and cDNA Biotin System (NuGen). Labelled cDNA was hybridized to Affymetrix GeneChip HT HG-U133+ PM Array plates. After washing and staining cell files were produced from scanned images (High Throughput Array Plate Scanner, Affymetrix) using Affymetrix Control Console software.

### ELISA

Cell-free BAL fluid and plasma from healthy controls and ARDS patients were assayed for human IL-1R2 using a Duo-Set ELISA (R&D Systems, Minneapolis, MN) and S100A12 using a CircuLex EN-RAGE ELISA Kit (MBL International, Woburn, MA). Assays were performed in accordance with the manufacturers’ instructions.

### Statistical analysis

Statistical analyses of microarrays were performed using ArrayStudio Version 5.0 (Omicsoft, NC). Data were normalized using robust multichip average method and log2-transformed and assessed by probset intensity, principle component analysis, hierarchical cluster analysis and sample correlation. General linear model with proper contrast tests were used. Differential expressed genes were identified using following criteria: a fold change of at least 2, raw p-value < 0.01 and estimated least squares mean intensity greater than or equal to 4 for at least one group in the comparison. Corrected p values using the Benjamini-Hochberg method were generated through the use of IPA (Ingenuity® Systems, www.ingenuity.com) to control for false discovery. Pathway and network analyses were also generated through the use of IPA.

Plasma and BAL IL-1R2 and S100A12 ELISA data were Log10 transformed and/or range scaled, as required, to achieve normal distribution for the performance of parametric statistical tests. The mean normalized concentrations of IL-1R2 and S100A12 in healthy controls were compared to the respective mean concentrations of these cytokines in ARDS patients using a one-way analysis of variance followed by a Dunnett’s post-test for multiple comparisons. The strength of associations between ARDS patient plasma and BAL cytokine concentrations and clinical phenotype data (e.g., APACHE III scores) were determined by logistic regression analysis. Statistical analyses were performed using GraphPad Prism 6.0 software for Windows (GraphPad Software, San Diego, CA).

## Results

### Inflammatory gene expression is increased in alveolar macrophages of ARDS patients

**Figure 1:**
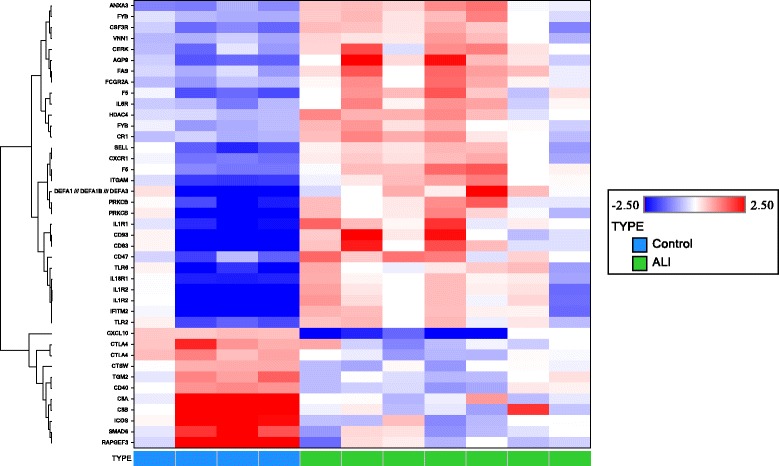
**Heat map for selected immune response genes expressed in isolated alveolar macrophages from patients with ARDS vs. healthy control subjects.** Each row represents a gene and each column represents a sample. Patients are classified as control (blue) or ARDS (green) as indicated at the bottom of the heat map. Red and blue indicate expression levels above and below the median, respectively. The dendrogram to the left of the matrix represents overall similarities in gene expression profiles.

Microarray analysis was performed on AM isolated from BAL fluid of patients with ARDS and controls. As compared to AM from non-diseased healthy control subjects, 229 probe sets were significantly up-regulated, whereas 344 probe sets were down-regulated in ARDS patients. A heat map of representative immune response genes is shown in Figure 1. The top up- and down-regulated genes observed in ARDS patients are shown in Table 3. Over-represented genes are primarily related to inflammatory, immune and injury responses, phagocytosis, and communication between innate and adaptive immune cells. The most highly expressed gene in the AM of ARDS patients was the leukocyte chemoattractant, S100A12.

**Table 3:** **Top up- and down-regulated genes in alveolar macrophages from ARDS patients as compared to controls**

**Gene**	**Fold increase (ARDS vs. Control)**	**Gene**	**Fold decrease (ARDS vs. Control)**
S100a12	49.43	FABP4	−40.62
IL-1R2	35.5	RND3	−37.53
CD177	34.32	ITIH5	−24.76
CRISPLD2	27.88	C8a	−22.89
OLFM4	25.24	FAM3B	−21.14
HLA-DQA1	23.6	NALCN	−20.03
Arginase1	19.16	SLC47α1	−19.12
IL-18R1	18.47	FHL1	−18.81
HLA-DRB1	18.09	VGLL3	−18.38
CYP1B1	17.68	HLA-DQB2	−17.83
MMP8	17.41	APO-C2	−16.77
ORM1	17.39	OASL	−15.1
KCNJ15	17.33	HLA-DRB4	−14.85
Annexin A3	17.18	SERPIN-G1	−14.63
STEAP4	17.16	RBP4	−13.8
MRVI1	17.11	C8b	−13.21
DYSF	16.47		
IL-8RA	16.06		
MCTP2	15.56		
MMP25	14.59		

**Figure 2:**
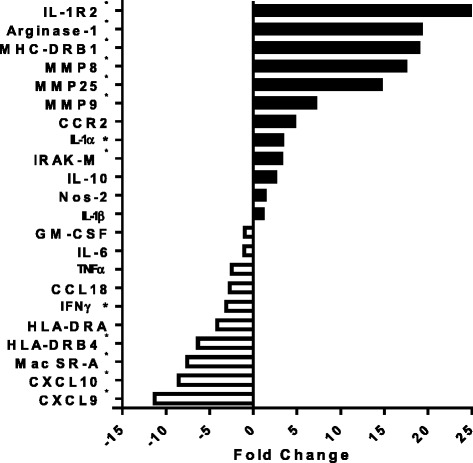
**mRNA expression of selected genes in alveolar macrophages of ARDS patients associated with macrophage activation states expressed as fold change compared to healthy control subjects.** Mean fold change was calculated from 4 healthy controls and 7 ARDS patients. (* p < 0.05 as compared to healthy subjects).

Data shown in Figure 2 represent up- and down-regulated genes that are associated with various macrophage activational states. Of the top up-regulated genes, several are characteristically expressed by alternatively activated macrophages, including arginase-1 (19.2-fold increase, p < 0.05), MHC Class II molecules (MHC-DRB1, 17.2-fold increase, p < 0.01), CCR2 (4.7-fold increase, p = 0.07), and several of the matrix metalloproteinases, including MMP8 (17.4-fold increase, p < 0.01), MMP9 (7.1-fold increase, p < 0.01) and MMP25 (14.6-fold increase, p < 0.01). Conversely, there was no significant change (TNF-α, IL-6, nos-2) or a reduction (IFN-γ, and the interferon-γ-inducible chemokines CXCL9 and CXCL10; 3.1-, 11.3-, and 8.6-fold decrease respectively, p < 0.05 for all) in the expression of genes associated with classical or M1 macrophage activation. We also observed preferential expression of genes associated with macrophage deactivation or immune tolerance in ARDS macrophages. Specifically, there was significantly increased expression of IRAK-3 (3.2-fold increase, p < 0.01, also referred to as IRAK-M) and trend toward enhanced IL-10, and reduced expression of HLA-DRA. One of the most highly expressed genes in ARDS macrophages was IL-1R2 (35.5-fold increase, p < 0.01). IL-1R2 is a non-signaling decoy receptor that functions to dampen IL-1 mediated cell activation [32].

### Gene expression in circulating buffy coat leukocytes of ARDS patients

**Figure 3:**
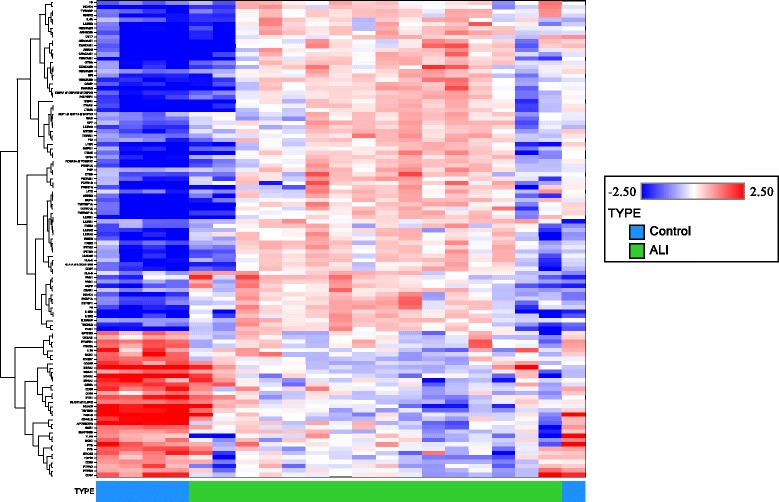
**Heat map for selected immune response genes expressed in buffy coat leukocytes from patients with ARDS vs. healthy control subjects.** Each row represents a gene and each column represents a sample. Patients are classified as control (blue) or ARDS (green) as indicated at the bottom of the heat map. Red and blue indicate expression levels above and below the median, respectively. The dendrogram to the left of the matrix represents overall similarities in gene expression profiles.

mRNA microarray analysis was also performed on buffy coat cells isolated from peripheral blood of patients with ARDS and healthy controls. As compared to control buffy coat cells, 1885 probe sets were significantly up-regulated and 2017 probe sets were significantly down-regulated in ARDS samples. Gene groups identified as up-regulated included those associated with leukocyte activation, cellular growth and proliferation, cellular metabolism (particularly oxidative phosphorylation), and B-cell, T-cell, and NK-cell signaling pathways. A representative heat map is shown in Figure 3.

The top up- and down-regulated genes expressed by circulating leukocytes from ARDS patients are shown in Table 4. Several members of the carcinoembryonic antigen-related cell-adhesion molecule (CEACAM) family are notably increased, namely CEACAM1, −6, and −8, members of the immunoglobulin subfamily which are associated with bacterial adhesion and cellular invasion. Additionally, several neutrophil-derived antimicrobial peptides are up-regulated, including alpha defensins, human bactericidal/permeability-inducing protein (BPI), and the cathelicidin CAMP.

**Table 4:** **Top up- and down-regulated genes in buffy coats from ARDS patients as compared to controls**

**Gene**	**Fold increase (ARDS vs. Control)**	**Gene**	**Fold decrease (ARDS vs. Control)**
CEACAM6	24.78	CD28	−11.9
Defensin α-4	23.78	HDAC9	−8.64
Defensin α-1	23.52	CRTAM	−8.06
CEACAM1	22.81	SERPINB9	−7.96
FCER1G	18.52	MSRA1	−6.85
CD14	18.45	TGFBR3	−6.55
ITGB2	14.86	GPR183	−6.45
THBS1	14.77	KLRC1/2	−6.19
BPI	14.18	CD96	−6.17
IFITM3	14.08	PTPRC	−4.94
ARRB2	13.92	FAS Ligand	−4.73
ITGB2	13.74	PRKRA	−4.28
NCF4	13.61	ETS1	−4.06
VNN1	12.14	GZMA	−3.91
FCGR1 Family	12.08	IL-15	−3.56
LILRA2	11.21		
PGLYRP1	11.09		
CAMP	10.45		
CEACAM8	10.34		

### Over- and underrepresented gene sets common to both alveolar macrophages and peripheral blood buffy coat cells

A total of 48 known genes were significantly overexpressed and 18 genes down-regulated in both ARDS macrophages and buffy coat cells as compared to control subjects (Table 5). Common upregulated genes included genes encoding chemotactic factors, antimicrobial peptides, chemokine receptors and matrix metalloproteinases. Of these up-regulated genes, S100A12, CD177, olfactomedin 4, and MMP8 were the most highly expressed in both macrophages and buffy coats of ARDS patients. Notably, the anti-inflammatory gene encoding IL-1R2 was also markedly over-expressed in both cell populations during ARDS.

**Table 5:** **Top up- and down-regulated genes common to both alveolar macrophages and buffy coats from ARDS patients as compared to controls**

**Gene symbol**	**Gene name**	**ARDS vs. Control fold change**	
		**Alveolar macrophages**	**Buffy coat**
S100A12	S100 calcium binding protein A12	49.43	5.56
IL1R2	Interleukin 1 receptor, type II	35.5	9.66
CD177	CD177 molecule	34.32	44.84
MGAM	Maltase-glucoamylase (alpha-glucosidase)	26.87	3.34
OLFM4	Olfactomedin 4	25.24	28.11
MMP8	Matrix metallopeptidase 8	17.41	31.96
ANXA3	Annexin A3	17.18	3.67
MRVI1	Murine retrovirus integration site 1 homolog	17.11	3.51
DYSF	Dysferlin	16.47	14.24
CXCR1	Chemokine (C-X-C) receptor 1	16.06	4.66
PROK2	Prokineticin 2	15.09	2.79
MMP25	Matrix metallopeptidase 25	14.59	12.52
METTL7B	Methyltransferase-like 7B	11.88	6.4
DEFA1	Defensin, alpha 1	11.43	23.52
GPR97	G protein-coupled receptor 97	11.07	9.29
F5	Coagulation factor V (proaccelerin)	9.79	6.45
LRG1	Leucine-rich alpha-2-glycoprotein 1	9.08	4.89
ADAM8	ADAM metallopeptidase domain 8	8.94	2.73
CSF3R	Colony stimulating factor 3 receptor	8.85	7.88
ARAP3	ArfGAP with RhoGAP domain, ankyrin repeat and PH domain 3	8.75	4.51
RASSF2	Ras association (RalGDS/AF-6) domain family member 2	8.51	8.01
PADI2	Peptidyl arginine deiminase, type II	7.86	12.49
FGF13	Fibroblast growth factor 13	7.35	5.91
RNF175	Ring finger protein 175	7.17	2.96
PRKCB	Protein kinase C, beta	7.01	7.02
PXN	Paxillin	6.34	6.01
VNN1	Vanin 1	6.07	10.24
GAS7	Growth arrest-specific 7	5.28	3.5
TSNAX	Translin-associated factor X	5.26	4.42
IFITM2	Interferon induced transmembrane protein 2 (1-8D)	5.21	9.44
GGT1	Gamma-glutamyltransferase 1	4.55	7.3
SIGLEC5	Sialic acid binding Ig-like lectin 5	4.45	7.57
BASP1	Brain abundant, membrane attached signal protein 1	4.2	2.8
MXD1	MAX dimerization protein 1	3.93	3.17
LMNB1	Lamin B1	3.78	4.13
CR1	Complement component (3b/4b) receptor 1	3.62	4.51
PSTPIP2	Proline-serine-threonine phosphatase interacting protein 2	3.37	2.9
GGTLC1	Gamma-glutamyltransferase light chain 1	3.28	3.3
PGS1	Phosphatidylglycerophosphate synthase 1	3.22	5.24
S100A8	S100 calcium binding protein A8	2.97	5.98
AQP9	Aquaporin 9	2.83	4.11
SLC22A4	Solute carrier family 22 (organic cation/ergothioneine transporter), member 4	2.83	3.17
IL17RA	Interleukin 17 receptor A	2.71	3.32
HDAC4	Histone deacetylase 4	2.66	4.25
ITGAM	Integrin, alpha M	2.58	2.47
AGTRAP	Angiotensin II receptor-associated protein	2.22	5.61
SMAP2	Small ArfGAP2	2.22	4.96
HLX	H2.0-like homeobox	2.17	4.84
IMMP2L	IMP2 inner mitochondrial membrane peptidase-like	−2.06	−3.08
L3MBTL3	l(3)mbt-like 3 (Drosophila)	−2.06	−2.34
CD3G	CD3g molecule, gamma (CD3-TCR complex)	−2.35	−2.47
PIGL	Phosphatidylinositol glycan anchor biosynthesis, class L	−2.4	−2.6
PLA2G16	Phospholipase A2, group XVI	−2.68	−2.3
CCDC50	Coiled-coil domain containing 50	−2.71	−4.71
ICA1L	Islet cell autoantigen 1,69 kDa-like	−3.07	−3.74
ZNF124	Zinc finger protein 124	−3.11	−4.54
WWOX	WW domain containing oxidoreductase	−3.39	−3.81
EPB41L4A	Erythrocyte membrane protein band 4.1 like 4A	−3.41	−10.19
RORA	RAR-related orphan receptor A	−4.17	−7.89
YES1	v-yes-1 Yamaguchi sarcoma viral oncogene homolog 1	−4.19	−4.55
RORA	RAR-related orphan receptor A	−4.21	−3.43
ZNF846	Zinc finger protein 846	−4.54	−2.31
SASH1	SAM and SH3 domain containing 1	−4.73	−4.29
ENPP5	Ectonucleotide pyrophosphatase/phosphodiesterase 5 (putative function)	−5.72	−4.99
JAKMIP2	Janus kinase and microtubule interacting protein 2	−5.77	−2.77
TCEA3	Transcription elongation factor A (SII), 3	−7.57	−2.77

### Determination of plasma and BAL fluid levels of IL-1R2 and S100A12 protein levels

**Figure 4:**
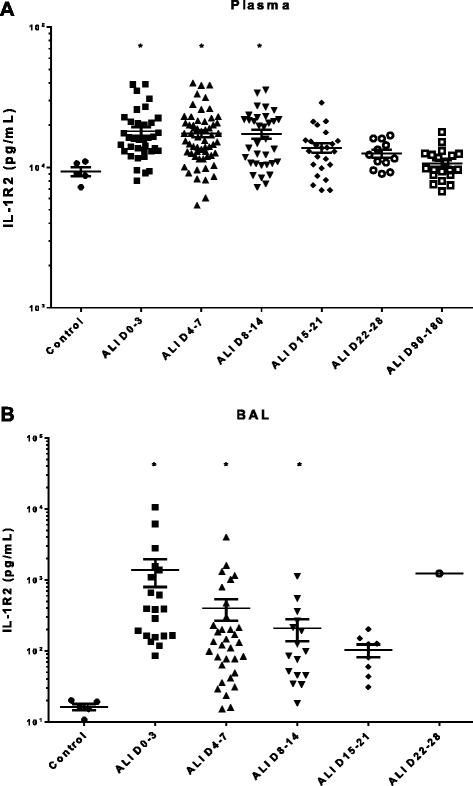
**Expression of IL-1R2 protein in (A) plasma samples and (B) BAL fluid of ARDS patients vs. healthy control subjects.** Protein levels were quantified by ELISA. Column means were analyzed by ANOVA with Dunnett’s multiple comparisons test. (*p < 0.05 as compared to healthy subjects).

**Figure 5:**
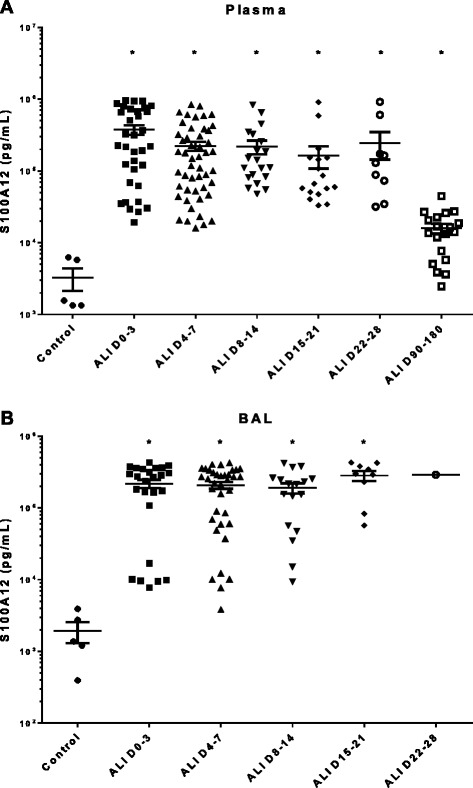
**Expression of S100A12 protein in (A) plasma and (B) BAL fluid of ARDS patients vs. healthy control subjects.** Protein levels were quantified by ELISA. Column means were analyzed by ANOVA with Dunnett’s multiple comparisons test. (*p < 0.05 as compared to healthy subjects).

We elected to focus on the top two up-regulated genes in AM, IL-1R2 and S100A12, to confirm our microarray findings. Protein levels of these two molecules in both plasma and cell-free BAL fluid were analyzed by ELISA in ARDS patients and healthy control subjects. IL-1R2 was significantly elevated in both the BAL fluid and plasma of ARDS patients compared with healthy controls. This increase was evident early in the course of ARDS (days 0–3), and persisted up to 8–14 days after the onset of disease (Figure 4). S100A12 was also significantly increased early in both the plasma and BAL of ARDS patients (days 0–3) and remained elevated in plasma for up to 90–180 days (Figure 5A), and in BAL fluid for up to 15–21 days (Figure 5B).

### Correlation of biomarker levels to patient demographic data

**Figure 6:**
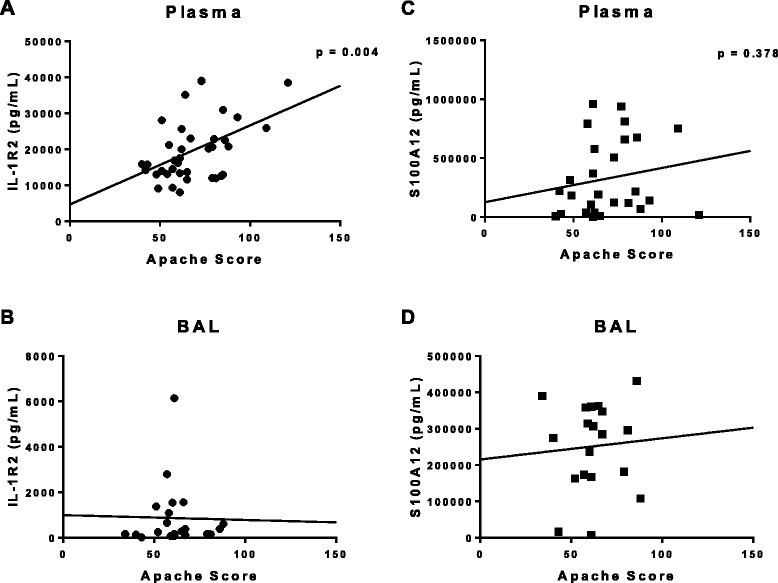
**Linear regression of plasma and BAL fluid IL-1R2 (A-B) and S100A12 (C-D) levels of ARDS patients within day 0–3 of disease onset as compared to APACHE score at initial presentation.** (Linear regression slope is considered significant if p < 0.05).

**Figure 7:**
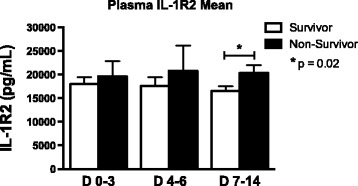
**Mean plasma IL-1R2 levels in ARDS survivors and non-survivors at in days 0–3, days 4–6, and days 7–14 post ARDS onset.** Column means were analyzed by ANOVA with Dunnett’s multiple comparisons test. (*p < 0.05 in non-survivors as compared to survivors).

Based on the observation that both IL-1R2 and S100A12 were significantly elevated in both plasma and BAL fluid of ARDS patients as compared with healthy control subjects, we compared levels of these molecules within ARDS patients to demographic and clinical outcomes data. Plasma IL-1R2 levels measured early (days 0–3) in the onset of disease significantly correlated with the APACHE III scores at the time of presentation to the hospital (Figure 6A, p < 0.005, R^2^ value 0.2134). Although plasma S100A12 levels increased with increasing APACHE scores, this correlation did not meet the level of statistical significance (Figure 6B). Neither BAL IL-1R2 nor S100A12 were significantly correlated with the initial APACHE score. Interestingly, we also observed a significant positive correlation between plasma IL-1R2 measured at days 7–14 and initial APACHE III score. Mortality in this study was low (<25%), Importantly, although, we did not observe significant associations between plasma or BAL IL-1R2 levels and survival in early ARDS,(peroids between days 0–3 or 4–6) , we found that mean plasma IL-1R2 levels measured between days 7–14 post ARDS onset were significantly higher in non-survivors as compared to survivors (Figure 7).

Of note, we did not observe any statistically significant correlations between either plasma or BAL biomarker levels and other clinical phenotypic data including PaO_2_/FIO_2_ (P/F) ratio, ventilator-free days, or organ failure-free days (Additional file 1: Figure S1, Additional file 2: Figure S2 and Additional file 3: Figure S3).

## Discussion

In this study, we sought to determine alterations in gene expression in leukocyte populations of patients with ARDS. In AM isolated from ARDS patients, many of the most highly up-regulated genes are involved in inflammation and immune responses to injury. CD177, which was also up-regulated in both the AM and in buffy coat cells of ARDS patients, is a molecule expressed by both activated neutrophils and macrophages that interacts with the PECAM-1 (platelet endothelial cell adhesion molecule 1), indicating a role in leukocyte extravasation and transmigration into inflammatory environments [33]. We also identified genes expressed in both AM and buffy coat cells during ARDS that have not previously been associated with pulmonary inflammation or injury. One such gene is olfactomedin 4, a protein expressed in bone marrow cells, tumor cells, and intestinal epithelium but found to be highly over-represented in our ARDS patient samples [34].

Although it was not unanticipated that we would observe an up-regulation of immune activation genes, it is notable that we also found a gene expression pattern in ARDS macrophages that displayed features of alternative activation or immune tolerance/deactivation. For example, arginase-1 was highly expressed in the AM of ARDS patients, and is a well-known product of alternatively AM. MMP8, −9 and −25 were also significantly up-regulated, and are associated with an alternative activation phenotype. There were also reciprocal decreases in genes that drive or are associated with classical M1 macrophage activation, including IFN-γ and IFN-γ-dependent CXC chemokines CXCL9 and CXCL10. Additionally, macrophages from ARDS patients displayed a molecular signature of immune tolerance/deactivation. For example, we observed no significant change or a trend toward reduced expression of important NF-κB-dependent pro-inflammatory cytokines, including TNF-α, IL-6, and IL-1β, whereas there was enhanced expression of the toll-like receptor signaling inhibitor IRAK-3 (IRAK-M) and a trend toward increased IL-10 message. In addition to suppression of a variety of inflammatory genes (tolerizable genes), an additional feature of LPS-tolerized macrophages is the superinduction of non-tolerizable genes that are primarily involved in host defense, including antimicrobial peptides [15]. This pattern is mimicked in both AM and especially buffy coat cells isolated from ARDS patients.

As noted previously, we observed alterations in gene expression suggesting a hybrid state of AM activation, with features of both alternative activation and immune tolerance/deactivation. This phenotype has not been described previously in lung macrophages during ARDS. It is possible that the phenotype observed represents a heterogenous population of macrophages within the lung. There is accumulating evidence of mixed macrophage phenotypes in other disease states, particularly in chronic inflammatory diseases [10,20-22]. It is not surprising that macrophages would display a healer M2 phenotype within the first 96 h post disease onset, which is consistent with the notion that lung fibroproliferation and repair are initiated early in the course of ARDS [35].

In addition to AM, we analyzed gene expression within circulating leukocytes of patients with ARDS. This population had a much broader range of differentially expressed genes, as compared with the AM, which is not unexpected given a more diverse cell population present in peripheral blood buffy coats. Canonical pathways that were activated in ARDS buffy coat cells included those involved in T-cell, B-cell, and NK-cell signaling, suggesting generalized lymphocyte activation. Additionally, pathways involved in leukocyte phagocytosis were over-represented, suggesting activation of circulating phagocytes. Furthermore, there were increases in integrin signaling and leukocyte extravasation signaling, reflective of the known migration of activated leukocytes to areas of inflammation.

Of potential genes that were up-regulated in AM and circulating leukocyte of ARDS patients, we chose to focus on two of the most highly induced genes, the proinflammatory cytokine S100A12 and the anti-inflammatory decoy receptor IL-1R2. We found that the S100 protein, S100A12, was the most highly expressed gene in lung macrophages from ARDS patients compared to controls. Notably, we also observed increases in S100A12 gene expression by circulating leukocytes of ARDS patients. This mRNA expression pattern paralleled the increased protein expression of S100A12 observed in both plasma and BAL fluid of ARDS patients. S100A12 is a known chemoattractant with an affinity for both neutrophils and monocytes, and has been shown to be a ligand for the RAGE receptor [24]. Although constitutively expressed in neutrophils [36], its expression in monocytes/macrophages is induced by early response molecules such as lipopolysaccharide (LPS) and TNFα.[37] Our observations are consistent with the previously published findings of increased S100A12 protein in both the BAL fluid and plasma of patients with ARDS [25-27]. While recognizing that S100A12 is up-regulated in plasma and BAL fluid of ARDS patients, these studies did not assess whether meaningful correlations could be made between levels of S100A12 and ARDS severity. We did not identify significant associations between this analyte and clinically relevant outcomes such as mortality, which may be due to the relatively small sample size and low observed mortality rate..

In addition to S100A12, we also observed strong mRNA induction of the IL-1 decoy receptor, IL-R2, in lung macrophages and buffy coat cells. IL-1R2 has a short cytoplasmic domain lacking the Toll/IL-1R domain required for intracellular signaling [38]. IL-1R2 regulates the proinflammatory activities of IL-1 by competitively binding to IL-1 at the cell surface and by sequestering IL-1R accessory protein and inhibiting the formation of a heterodimeric signaling complex with IL-1R1 [39]. In response to proinflammatory stimuli such as LPS or TNFα, proteolytic cleavage of the membrane-bound portion of IL-1R2 occurs, resulting in a soluble form which is capable of sequestering IL-1 at sites of inflammation [28,40,41]. Although the molecule has been shown to be up-regulated in the setting of sepsis [28] as well as recently in a mouse model of chemically-induced lung injury [29], this gene has not previously been recognized as a biomarker candidate of disease severity in human ARDS. In this study, we have demonstrated for the first time that IL-1R2 is significantly increased in both plasma and BAL fluid of ARDS patients as compared to healthy subjects. Furthermore, we have shown that plasma IL-1R2 levels measured both early (within 96 h) and late (between days 7–14) after the onset of ARDS significantly correlated with APACHE scores at the time of presentation to the hospital. Collectively, these findings suggest that IL-1R2 reliably reflects the severity of systemic illness in ARDS patients. Based on the well-established association between APACHE score and mortality in critical illness [42], it is not unreasonable to suspect that plasma IL-1R2 levels may be predictive of mortality in ARDS patients. Although we did not see a significant correlation between plasma IL-1R2 levels and mortality in the early stages of ARDS (≤6 days post onset), we did observe that mean plasma IL-1R2 levels obtained later in the course of ARDS (between days 7–14 post onset) were significantly higher in non-survivors as compared to survivors, which supports an association between persistently high plasma IL-1R2 levels and increased mortality. A larger study would be required to detect correlations between IL-1R2 levels and other clinically significant outcomes.

Our study has several limitations. Importantly, our microarray analysis was limited to a single time point, which was relatively early after the onset of ARDS (<96 h). Moreover, the use of healthy subjects as a control group is not optimal. Because of the small sample size, we were unable to correlate changes in gene expression patterns with causes of ARDS or certain clinical outcomes. However, we were able to verify expression of protein corresponding to selected over-expressed genes in both plasma and BAL fluid, with a sample size large enough to show a significant correlation to illness severity. Additionally, we have shown for the first time the plasma IL-1R2 levels are elevated in ARDS patients, and persistent elevations of this biomarker correlate with increased mortality.

## Conclusions

Taken together, our results show that both S100A12 and IL-1R2 are potential biomarkers of ARDS that are present in both the lung airspace and in circulation. This pilot study provides important insights into ARDS pathogenesis, including the identification of pathogenic molecules and potential novel biomarkers in this disease.

## Additional files

Additional file 1: Figure S1. Linear regression of plasma and BAL fluid IL-1R2 (A-B) and S100A12 (C-D) levels of ARDS patients within day 0–3 of disease onset as compared to PaO_2_/FIO_2_ ratios (P/F) at initial presentation. Additional file 2: Figure S2. Linear regression of plasma and BAL fluid IL-1R2 (A-B) and S100A12 (C-D) levels of ARDS patients within day 0–3 of disease onset as compared to ventilator-free days (VFD). Additional file 3: Figure S3. Linear regression of plasma and BAL fluid IL-1R2 (A-B) and S100A12 (C-D) levels of ARDS patients within day 0–3 of disease onset as compared to organ failure-free days (OFD).

## Abbreviations

ARDS: Acute respiratory distress syndrome
M1: classically activated macrophage
M2: alternatively activated macrophage
PAMP: pathogen associated molecular pattern
LPS: lipopolysaccharide
IFNγ: interferon gamma
TNFα: tumor necrosis factor alpha
NO: nitric oxide
ROS: reactive oxygen species
IL: interleukin
S100A12: S100 calcium binding protein A12
RAGE: receptor for advanced glycation endpoints
BAL: bronchoalveolar lavage fluid
IL-1R2: interleukin-1 receptor 2
AM: alveolar macrophage
APACHE III: Acute physiology and chronic health evaluation III
GMCSF: Granulocyte-macrophage colony stimulating factor
ELISA: Enzyme-linked immunosorbent assay
CEACAM: Carcinoembryonic antigen-related cell adhesion molecule
BPI: Bactericidal/permeability-inducing protein
CAMP: Cathelicidin antimicrobial peptide
PECAM-1: Platelet endothelial cell adhesion molecule 1
