# Supplementary figures and images for: Microarray analysis identifies IL-1 receptor type 2 as a novel candidate biomarker in patients with acute respiratory distress syndrome
Source: Respir Res. 2015 Feb 21;16(1):29. doi: 10.1186/s12931-015-0190-x (PMC4339297; doi:10.1186/s12931-015-0190-x)

## Slide 1
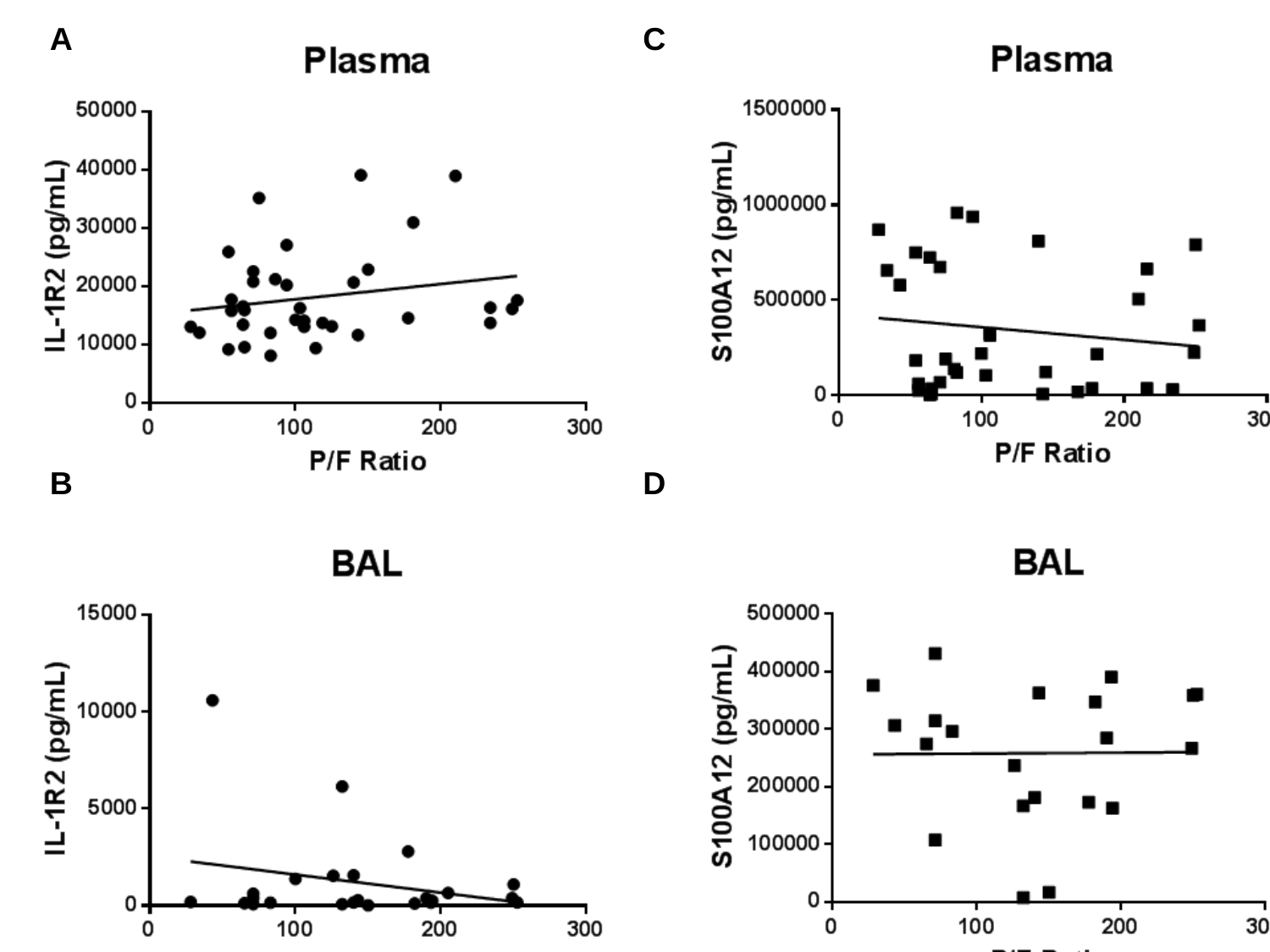

A
C
B
D

Supplement: Additional file 1: Figure S1. — Linear regression of plasma and BAL fluid IL-1R2 (A-B) and S100A12 (C-D) levels of ARDS patients within day 0–3 of disease onset as compared to PaO2/FIO2 ratios (P/F) at initial presentation. [file 12931_2015_190_MOESM1_ESM.pptx]

## Slide 1
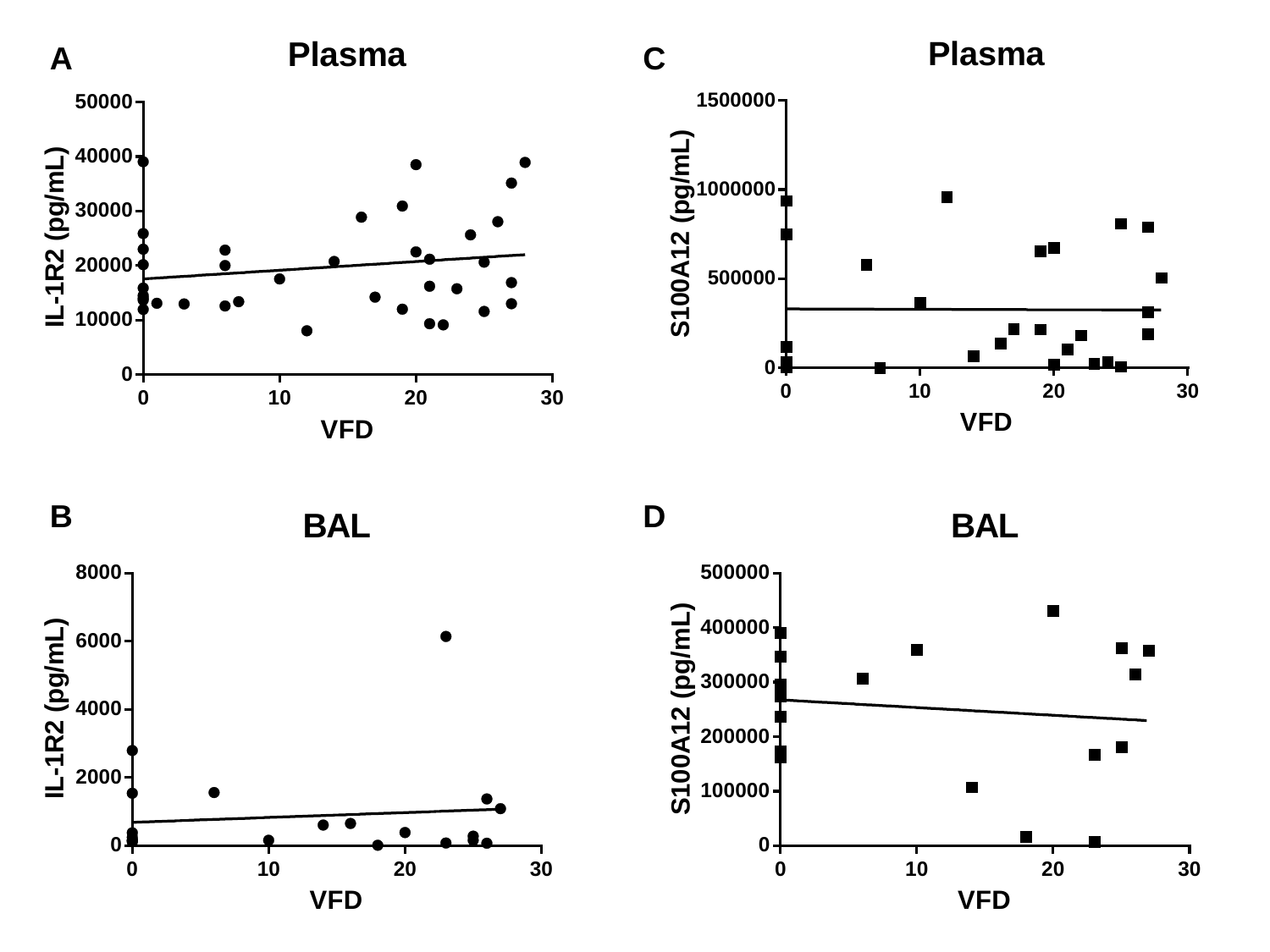

A
C
B
D

Supplement: Additional file 2: Figure S2. — Linear regression of plasma and BAL fluid IL-1R2 (A-B) and S100A12 (C-D) levels of ARDS patients within day 0–3 of disease onset as compared to ventilator-free days (VFD). [file 12931_2015_190_MOESM2_ESM.pptx]

## Slide 1
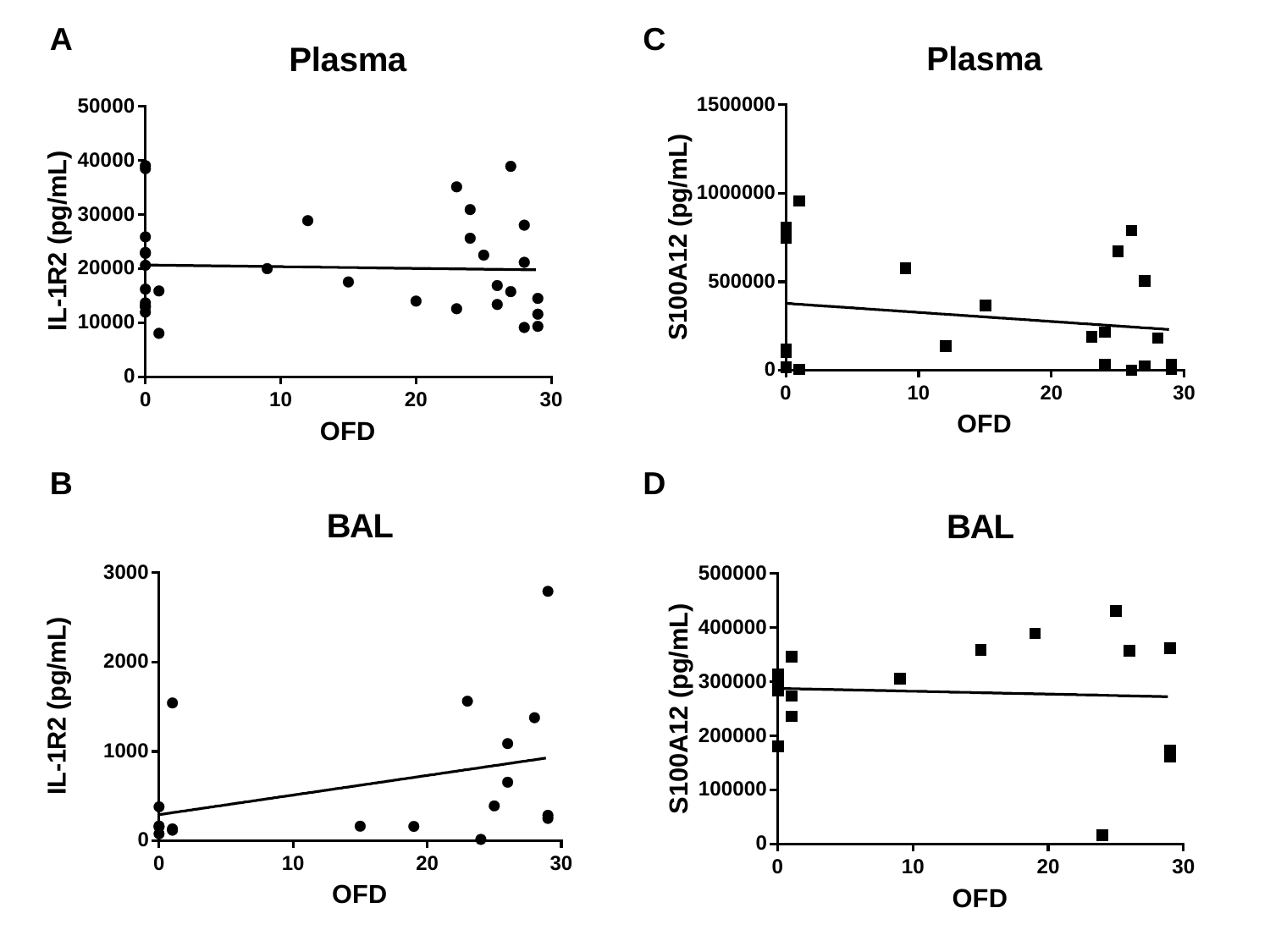

A
C
B
D

Supplement: Additional file 3: Figure S3. — Linear regression of plasma and BAL fluid IL-1R2 (A-B) and S100A12 (C-D) levels of ARDS patients within day 0–3 of disease onset as compared to organ failure-free days (OFD). [file 12931_2015_190_MOESM3_ESM.pptx]
